# Development, High-Throughput Profiling, and Biopanning of a Large Phage Display Single-Domain Antibody Library

**DOI:** 10.3390/ijms25094791

**Published:** 2024-04-27

**Authors:** Hee Eon Lee, Ah Hyun Cho, Jae Hyeon Hwang, Ji Woong Kim, Ha Rim Yang, Taehoon Ryu, Yushin Jung, Sukmook Lee

**Affiliations:** 1 Department of Biopharmaceutical Chemistry, Kookmin University, Seoul 02707, Republic of Korea; hyun113@kookmin.ac.kr (H.E.L.); ahhyun0601@kookmin.ac.kr (A.H.C.); wo10gus@kookmin.ac.kr (J.H.H.); jwk7853@kookmin.ac.kr (J.W.K.); 2825760@kookmin.ac.kr (H.R.Y.); 2 ATG Lifetech Inc., Seoul 08507, Republic of Korea; thryu@atglifetech.com (T.R.); yushin.jung@atglifetech.com (Y.J.); 3 Department of Applied Chemistry, Kookmin University, Seoul 02707, Republic of Korea; 4 Antibody Research Institute, Kookmin University, Seoul 02707, Republic of Korea

**Keywords:** single-domain antibody, next-generation sequencing, antibody library, phage display

## Abstract

Immunoglobulin G-based monoclonal antibodies (mAbs) have been effective in treating various diseases, but their large molecular size can limit their penetration of tissue and efficacy in multifactorial diseases, necessitating the exploration of alternative forms. In this study, we constructed a phage display library comprising single-domain antibodies (sdAbs; or “VHHs”), known for their small size and remarkable stability, using a total of 1.6 × 10^9^ lymphocytes collected from 20 different alpacas, resulting in approximately 7.16 × 10^10^ colonies. To assess the quality of the constructed library, next-generation sequencing-based high-throughput profiling was performed, analyzing approximately 5.65 × 10^6^ full-length VHH sequences, revealing 92% uniqueness and confirming the library’s diverse composition. Systematic characterization of the library revealed multiple sdAbs with high affinity for three therapeutically relevant antigens. In conclusion, our alpaca sdAb phage display library provides a versatile resource for diagnostics and therapeutics. Furthermore, the library’s vast natural VHH antibody repertoire offers insights for generating humanized synthetic sdAb libraries, further advancing sdAb-based therapeutics.

## 1. Introduction

Immunoglobulin G (IgG)-based monoclonal antibodies (mAbs) have emerged as a highly promising therapeutic approach for treating a diverse range of diseases, encompassing inflammatory disorders, neurodegenerative diseases, cancers, and infectious diseases [1]. Their targeted mechanism of action and specificity have revolutionized the treatment of disease, significantly improving patient outcomes. However, the complexity of multifactorial diseases poses challenges to the therapeutic efficacy of existing mAbs, prompting the exploration of innovative strategies to enhance their effectiveness [2]. To address these unmet medical needs, single-domain antibodies (sdAbs), also known as VHHs, have gained global recognition as a versatile platform for generating a variety of therapeutic modalities [3].

Camelids, including alpacas, have three known IgG isotypes (IgG1, IgG2, and IgG3), among which, IgG2 and IgG3 are unique as heavy-chain-only antibodies (HCAbs). Single-domain antibodies (sdAbs), also known as VHHs, are derived from the variable domain of HCAbs found in camelids and lack the light chain (LC) components [4]. Similar to the variable heavy (VH) region of conventional antibodies, VHHs also possess three complementarity-determining regions (CDRs) and four framework regions (FRs) [5,6]. CDR1 and CDR2 are encoded by the V germline gene segment, whereas CDR3 is formed by V(D)J recombination [7,8]. VHHs exhibit several advantageous properties that make them attractive for diagnostic and therapeutic applications. One notable feature of VHHs is their smaller size (~15 kDa) compared with conventional antibodies [9,10]. This reduced size enhances their penetration of tissue, enabling more effective delivery to the target sites [11]. VHHs also exhibit remarkable stability when exposed to high temperatures for prolonged periods [12,13]. Another characteristic of VHHs is that they possess hydrophilic residues at four positions (IMGT numbers 42, 49, 50, and 52), which interact with the variable light (VL) region in the FR2 of the conventional VH region, conferring additional advantages for solubility [14,15,16,17]. Furthermore, VHHs demonstrate high specificity and affinity for their target antigens, making them potent therapeutic candidates [18]. Lastly, the single-domain nature of VHHs allows for flexible engineering into various recombinant antibody formats, facilitating the development of customized antibody-based therapeutics [19]. These unique attributes make VHHs valuable tools for a range of applications, including therapeutics as monospecific or multispecific antibodies, antibody–drug conjugates (ADCs) and chimeric antigen receptor T cells (CAR-T) as well as for diagnostics and research purposes [20,21,22,23,24]. Notably, VHH-based therapeutics have already received regulatory approval, such as caplacizumab, ciltacabtagene autoleucel, and ozoralizumab. Caplacizumab, a humanized bivalent single-domain antibody, is approved for the treatment of thrombotic thrombocytopenic purpura (TTP) and thrombosis [25]. Ciltacabtagene autoleucel, a B-cell maturation antigen (BCMA)-directed CAR-T, has received approval for treating patients with relapsed or refractory multiple myeloma [26]. Ozoralizumab, a trivalent anti-tumor necrosis factor alpha (TNFα) humanized single-domain antibody, is approved for the treatment of rheumatoid arthritis [27].

The antibody display system includes the phage display, yeast display, bacterial surface display, and ribosome display [28,29,30]. Among these systems, a phage display antibody library is a collection of diverse antibody fragments genetically fused to coat proteins on the surface of bacteriophages, allowing for the display of a wide range of antibodies [31,32,33,34]. It serves as a versatile and widely used tool for engineering antibodies and drug discovery [35]. The diversity of the library plays a pivotal role in isolating antibodies with high specificity for a given target using phage display technology. In mammals, V(D)J recombination is a key process that contributes to the diversification of the B cell receptor (BCR) repertoire, which is capable of recognizing a wide range of antigens [36]. Additionally, random nucleotide deletions or insertions at the junction regions of gene segments contribute to the final diversity of the BCR repertoire [37]. For naïve libraries constructed without any process of immunization, the theoretical diversity increases with the number of initially collected lymphocytes, enriching a more extensive range of potential binding specificities. Although it has been reported that a single donor is sufficient to produce a highly functional antibody library, recent studies of the B cell repertoire have suggested that individuals possess a unique immune system and do not extensively share common B cell clonotypes [20,38]. Hence, multiple donors’ blood samples are beneficial for expanding the antibody library’s repertoire. Therefore, we assumed that the construction of phage display libraries using numerous lymphocytes collected from an increased number of different donors would enhance the diversity of the library, leading to the rapid discovery of diverse high-quality antibodies.

Next-generation sequencing (NGS), also known as high-throughput sequencing, is an emerging technology for rapidly sequencing DNA fragments in large-scale experiments [39]. In contrast to the traditional Sanger sequencing method, which has limitations, such as intensive labor, high cost, and the limited throughput of the number of DNA types that can be decoded at once, this high-throughput capability is especially valuable for the characterization and assessment of the quality of antibody libraries [40,41]. NGS allows researchers to comprehensively analyze the diversity and distribution of antibody sequences within the library, providing insights into the richness of the immune repertoire [42].

In our study, we constructed a large sdAb phage display library using a total of 1.6 × 10^9^ lymphocytes obtained from 1 L of blood collected from 20 different non-immunized alpacas to ensure a diverse VHH repertoire. Our NGS analysis allowed us to comprehensively assess the vast array of VHH sequences present in the library, providing valuable insights into its potential for the discovery of antibodies. Furthermore, we utilized phage display technology to isolate VHHs that specifically target three therapeutically relevant antigens from the constructed library. Through the selection of VHHs with high binding affinity and specificity using phage display, we successfully identified potential hits for future therapeutic applications. Overall, the established alpaca VHH library, characterized using high-throughput NGS, will serve as a valuable resource for the rapid development of target-specific VHHs. These VHHs hold promise for use in various antibody-based therapeutic platforms.

## 2. Results

### 2.1. Construction of an Alpaca Single-Domain Antibody (VHH) Phage Display Library

**Figure 1:**
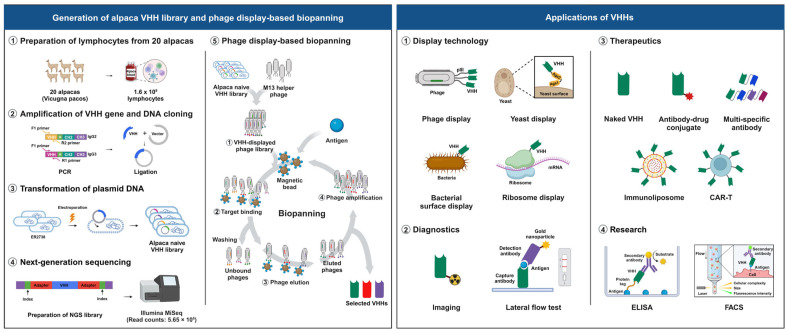
Schematic representation of the process of constructing an alpaca VHH library, phage display-based biopanning, and the applications of VHHs. The VHH library was constructed from 1.6 × 10^9^ alpaca lymphocytes, and VHHs were selected through phage display-based biopanning. VHHs hold promise for a wide range of applications, including display technology, diagnostics, therapeutics, and research.

To generate a large and diverse VHH library, a total of 1.6 × 10^9^ lymphocytes were obtained from 1 L of blood collected from 20 different non-immunized alpacas. Total RNA was extracted from the lymphocytes and reverse-transcribed into the complementary DNA (cDNA). The VHH genes were then amplified from the cDNA using polymerase chain reaction (PCR) with gene-specific primers targeting the VHH germline genes (Supplementary Table S1). The amplified VHH genes were cloned into the pComb3XSS phagemid DNA vector at the Sfi I sites. The resulting VHH library was introduced into *Escherichia coli* (*E. coli*) ER2738 using electroporation. The process of construction yielded a substantial number of VHH clones, resulting in a library size of approximately 7.16 × 10^10^ (Figure 1). On the basis of these results, the newly generated alpaca VHH phage display library is expected to contain a diverse repertoire of VHHs, making it a valuable resource for the isolation and selection of specific VHHs with high affinity and specificity for therapeutically relevant antigens.

### 2.2. NGS of the Constructed Alpaca VHH Phage Display Library

**Figure 2:**
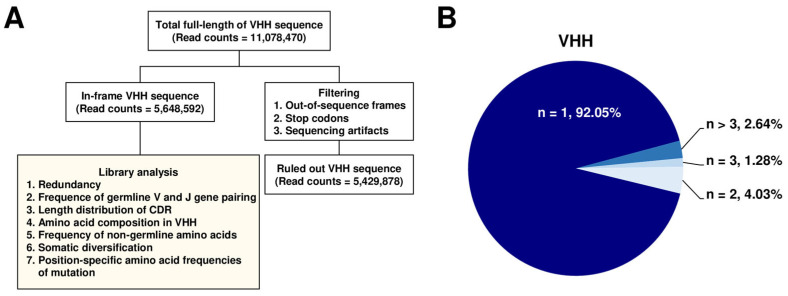
Next-generation sequencing (NGS)-based characterization of the constructed single domain antibody library. (**A**) Flowchart illustrating the process of NGS analysis. (**B**) Redundancy analysis of the variable regions (VHHs) of heavy-chain-only antibody sequences in the constructed library. The graph displays the number of replicates (n) as a percentage of VHH sequences out of the total number of analyzed sequences.

To explore the diversity of VHH repertoires within the constructed library, we conducted an NGS analysis. Initially, we isolated phagemid DNA sequences encoding the VHH clones from the *E. coli* transformants. Subsequently, VHH amplicon libraries were generated through PCR for NGS. The sequencing runs produced a total of 11,078,470 VHH reads. To ensure the accuracy and quality of the data, we performed several processing steps and filtering procedures to eliminate reads containing sequencing artifacts, stop codons, and out-of-sequence frames. After this filtering, we obtained a refined dataset of 5,648,592 reads for further analysis (Figure 2A).

To assess the redundancy of VHH domain sequences, we examined the presence of unique VHH clones in the dataset. Remarkably, approximately 92% of the VHH sequences appeared only once (Figure 2B). These results indicated that the constructed library predominantly comprised distinct and diverse VHH clones capable of isolating and characterizing a wide array of VHHs with potential applications in therapeutic development and diagnostics.

### 2.3. Analysis of Germline Usage in the Alpaca VHH Phage Display Library

**Figure 3:**
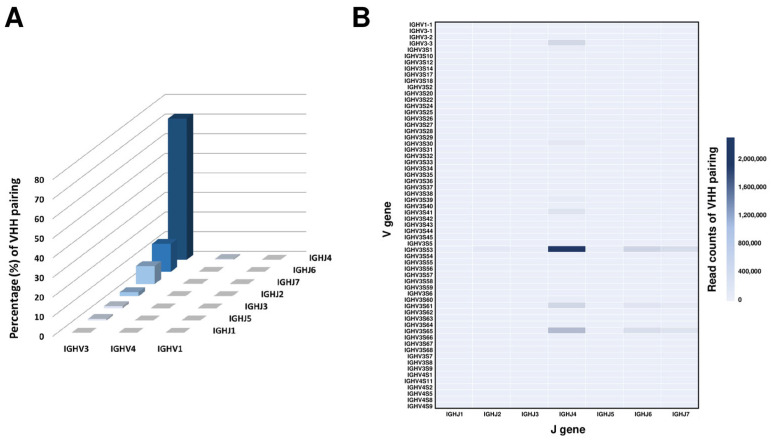
Germline V and J gene pairing frequencies in the VHH repertoire of the constructed library. (**A**) Visualization of the germline V and J gene pairing frequencies within the VHH repertoire of the constructed library. This two-dimensional heatmap depicts the frequencies of the pairings between the three IGHV gene families and the seven IGHJ gene families. (**B**) Further depiction in heatmap format, showcasing the frequencies of pairings between the 63 individual IGHV gene segments and the seven IGHJ gene families within the VHH sequences.

To investigate the usage of the VHH gene repertoire within the constructed library, a dataset of 5,648,592 VHH sequences underwent in-depth analysis using the alpaca genomic database of the international ImMunoGeneTics information system (IMGT) in conjunction with the IgBLAST analysis tool. Each VHH sequence was categorized on the basis of its alignment with the three IGHV and seven IGHJ families found within the VHH repertoire. Notably, a substantial portion of the sequences demonstrated alignment with specific IGHV and IGHJ family pairs. The three most prevalent pairs identified within the VHH family were IGHV3_IGHJ4 (72.0%), IGHV3_IGHJ6 (14.3%), and IGHV3_IGHJ7 (9.2%), observed in descending order of frequency (Figure 3A). This comprehensive analysis highlighted the pronounced dominance of the IGHV3 and IGHJ4 gene families.

To further confirm the overall trend of VJ recombination in the library, we analyzed the pairs and frequencies of individual 63 IGHV gene segments and 7 IGHJ gene subfamilies in VHH (Figure 3B). The most common VJ gene combinations in the VHH sequences, in descending order, were IGHV3S53_IGHJ4 (41.5%), IGHV3S65_IGHJ4 (12.0%), and IGHV3S53_IGHJ6 (6.0%). In addition, the analysis revealed that IGHV3S53 and IGHJ4 were the most frequently utilized gene segments among the IGHV and IGHJ gene families, respectively (Supplementary Table S2).

### 2.4. Analysis of CDRs and the FR2 Characteristics in Alpaca VHHs within the Constructed Library

**Figure 4:**
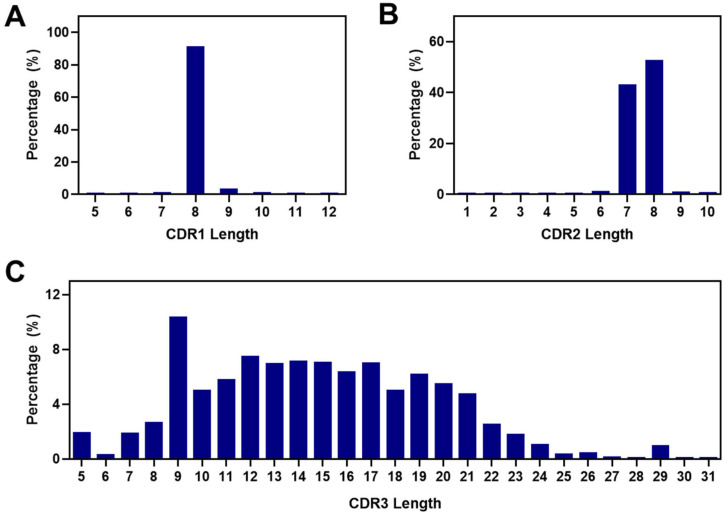
The length distribution of VHHs’ complementarity-determining regions (CDRs) in the constructed library. (**A**–**C**) Frequencies of the lengths of CDR1 (**A**), CDR2 (**B**), and CDR3 (**C**) expressed as percentage bar graphs. The most common length of CDR1 and CDR2 was eight amino acids. The lengths of CDR3 were found to range from 5 to 31 amino acids.

Analysis of the 5,648,592 VHH sequences showed that the predominant length of CDR1 in the VHH sequence was eight amino acids (Figure 4A). For CDR2, the most common lengths were eight and seven amino acids (Figure 4B). The length of CDR3 ranged from 5 to 31 amino acids. Among these lengths, a CDR3 length of nine amino acids was the most frequently observed (Figure 4C). The data indicated that CDR3 lengths ranging from 10 to 21 amino acids had an average occurrence of 6%, showing an even distribution overall.

**Figure 5:**
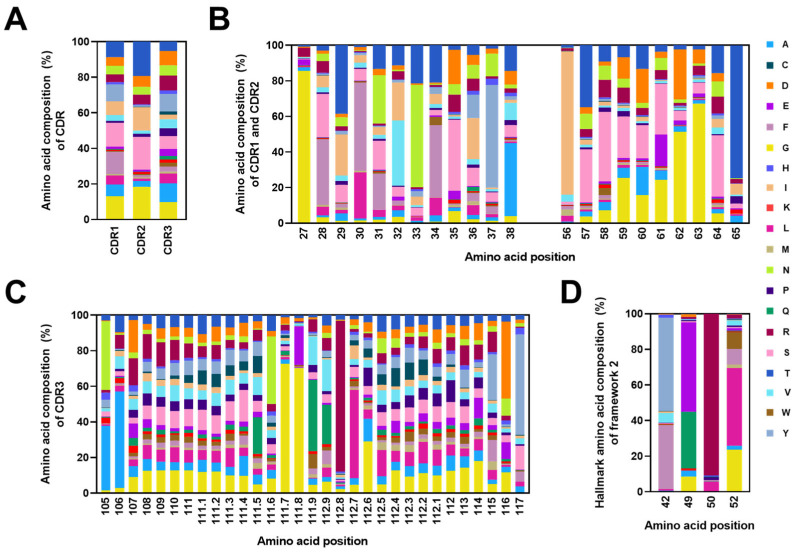
Amino acid compositions in VHH’s CDRs and Framework 2 (FR2). (**A**) Comprehensive analysis of the amino acid compositions across the complete CDR1, CDR2, and CDR3 within the constructed library. (**B**,**C**) In-depth investigation into the amino acid compositions at each position within CDR1, CDR2, and CDR3. (**D**) Detailed analysis of the characteristic amino acid compositions at Positions 42, 49, 50, and 52 within the FR2. In all panels (**A**–**D**), the amino acid compositions within CDRs and FR2 are expressed as percentages and visualized as stacked bars in distinct colors. Each stacked bar represents the proportion of each amino acid at specific positions. The *x*-axis indicates the amino acids’ positions in accordance with the IMGT’s numbering.

To assess the diversity in amino acid usage within the CDRs of the VHH repertoire, we analyzed the amino acid compositions in CDR1, CDR2, and CDR3 from the constructed library. For VHH, the amino acid compositions at each position in CDR1 (IMGT numbers 27–38), CDR2 (IMGT numbers 56–65), and CDR3 (IMGT numbers 105–117) were determined. The analysis revealed that CDR3 exhibited much higher diversity compared with CDR1 and CDR2 (Figure 5B,C). The amino acids alanine (10.6%), tyrosine (9.9%), glycine (9.7%), and arginine (8.3%) were frequently observed in CDR3 from the library. Interestingly, the presence of cysteine residues in CDR1 and CDR2 was negligible, accounting for only 0.14% and 0.16%, respectively (Figure 5A, Supplementary Tables S3 and S4).

Additionally, we examined the amino acid compositions in four positions (42, 49, 50, and 52) within the VHH’s Framework 2 (FR2). Consistent with previous studies, the hydrophilic residues were present in these positions. The predominantly observed amino acids were tyrosine (53.3%) and phenylalanine (35.1%) at Position 42, glutamic acid (49.2%) and glutamine (32.8%) at Position 49, arginine (90.7%) at Position 50, and leucine (44.7%) and glycine (22.5%) at Position 52 (Figure 5D). These results confirmed the presence of characteristic hydrophilic residues in the FR2 of VHH. This distinctive composition differed from that of conventional antibodies, which typically feature hydrophobic residues at these specific positions.

### 2.5. Frequencies of Mutations in CDRs

**Figure 6:**
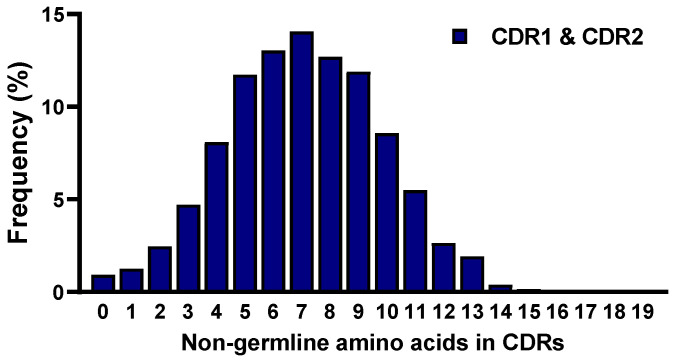
The frequency of non-germline amino acids within CDR1 and CDR2 in the constructed library. The graph depicts the percentage of VHH sequences with different numbers of non-germline amino acid residues in CDR1 and CDR2. The *x*-axis shows the number of non-germline amino acids in CDR1 and CDR2, while the *y*-axis indicates the percentage of VHH sequences. The data reveals the distribution of mutations in the CDRs, with some sequences exhibiting no mutations, and others displaying varying numbers of non-germline amino acids.

To assess the frequency of mutations in the constructed library, we compared the sequences of the VHH clones with their corresponding germline reference sequences. This analysis was performed by aligning the 5,648,592 VHH sequences with the most closely matched germline VHH gene segments in the IMGT alpaca antibody database using the IgBLAST analysis tool. Specifically, we focused on the mutations observed in the CDRs of the VHH sequences. The frequencies of non-germline amino acids in CDR1 and CDR2 were calculated by comparing the CDRs’ amino acid sequences from the obtained VHH with those of the reference germline. The results showed that 0.92% of the sequences of CDR1 and CDR2 had no mutations out of the 5,648,592 VHH clones. In addition, 88.4% of the sequences of CDR1 and CDR2 had 1 to 10 mutations, while the remaining 10.7% had more than 10 mutations. On average, seven amino acids were found to be mutated in CDR1 and CDR2 (Figure 6).

**Figure 7:**
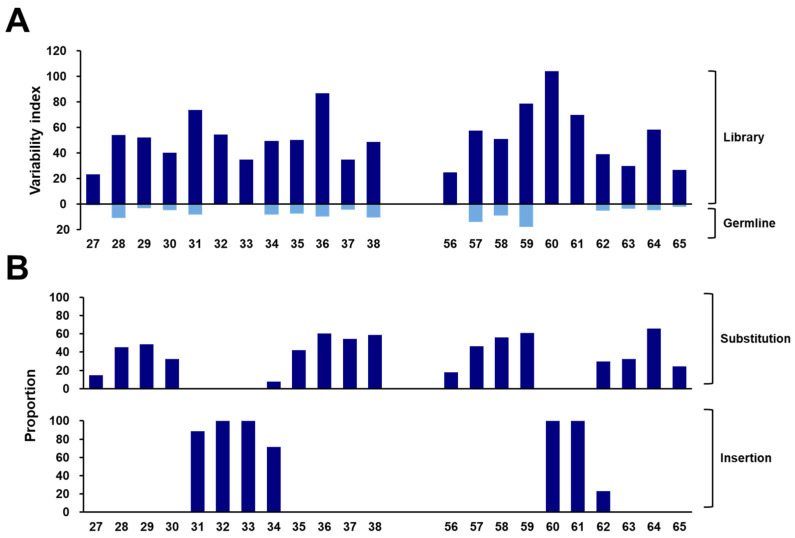
Somatic diversification in the constructed library. (**A**) Variability indexes of VHHs in the constructed library and the alpaca germline database. The variability index is shown as a bar graph, with the positions exhibiting high variability being marked. (**B**) Mutation (substitution and insertion) hotspots in alpaca VHH genes. The frequencies of mutations at each position within the CDRs are illustrated. The *x*-axis indicates the amino acids’ position for each CDR, as defined by the IMGT’s numbering.

To calculate the variability index at each position in the constructed library, we compared it with the germline sequences obtained from the IMGT database (Figure 7A). The variability index in the constructed library was significantly higher than that of the germline sequences, particularly those with values under 20. Notably, both CDR1 and CDR2 exhibited variability indices exceeding 20, with an even more pronounced elevation at five positions (31, 36, 59, 60, and 61) where the index exceeded 60, indicating a notable frequency of somatic mutations concentrated in these specific regions. Furthermore, we determined the frequencies of mutations encompassing substitutions and insertions within the CDRs (Figure 7B). The mutation frequencies were observed to be 45.1% and 44.3% in CDR1 and CDR2, respectively. Specifically, the positions where substitutions occurred most frequently were 36, 37, 38, 58, 59, and 64, each with over 50% frequency. Moreover, Positions 31, 32, 33, 34, 60, and 61 showed the highest frequency of insertions, with over 60%. Although the frequency was relatively low, insertions occurred at a rate of 23.1% even at Position 62. These findings substantiated the diverse spectrum of VHH antibodies present in the constructed library, with these positions contributing significantly to the repertoire’s richness.

**Figure 8:**
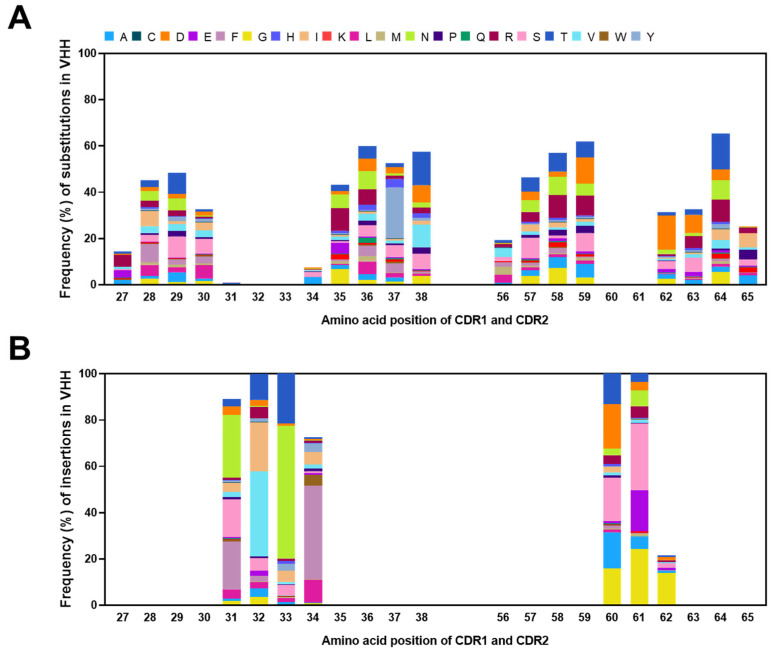
Position-specific amino acid frequencies of mutations at each position in the CDRs of the VHH in the constructed library. Position-specific amino acid frequencies of substitutions (**A**) and insertions (**B**) at each position in the CDRs of the variable domain in the constructed library. The *x*-axis indicates the amino acids’ position of each CDR, as defined by the IMGT’s numbering.

The amino acid composition of substituted residues at specific positions was determined in the VHH sequences (Figure 8). In CDR1 and CDR2, Position 64, which showed the highest frequency of mutation, had threonine (16.1%) as the amino acid with the highest mutation ratio, followed by aspartic acid (11.6%) at Position 59, asparagine (7.2%) at Position 36, and threonine (15.0%) at Position 38 (Figure 8A). Furthermore, we observed that the most frequently substituted amino acids in CDR1 and CDR2, in descending order, were threonine (11.9%), arginine (10.6%), and serine (10.0%). Additionally, the amino acid composition of the inserted residues at each CDR position was determined in the VHH sequences (Figure 8B). The most frequently inserted amino acid in CDR1 and CDR2 was glycine (36.5%).

### 2.6. Selection of Antigen-Specific VHH Antibodies from the Constructed Library

To validate the suitability of the constructed library as a high-quality antibody selection tool against the proteins of interest, we utilized biopanning, a widely used technique for isolating antigen-specific antibodies from phage display libraries. Biopanning was performed against three therapeutically significant antigens: programmed cell death ligand 1 (PD-L1) and the receptor-binding domains (RBDs) of two severe acute respiratory syndrome coronavirus 2 (SARS-CoV-2) variants, delta (B.1.617.2) and omicron (BA.2). After biopanning, 96 colonies were randomly picked and analyzed through phage enzyme-linked immunosorbent assay (ELISA), and sequencing efforts were conducted to identify the target-specific VHHs (Supplementary Figure S1). After narrowing down the overlapped sequences, we expressed and purified the selected VHH clones using affinity column chromatography. One clone specific to the RBD of SARS-CoV-2 omicron (BA.2) was excluded due to low expression levels in the *E. coli* system. In conclusion, we selected two VHHs specific to PD-L1, six VHHs specific to the RBD of SARS-CoV-2 delta (B.1.617.2), and three VHHs specific to the RBD of SARS-CoV-2 omicron (BA.2). In the selected VHHs, the most common length of both CDR1 and CDR2 was eight amino acids (Supplementary Table S5), while the length of CDR3 varied from 8 to 19 amino acids. The dominant VJ gene combinations observed in the selected VHHs, ranked in descending order, were IGHV3S53_IGHV (36.4%), IGHV3S1_IGHJ6 (27.3%), and IGHV3-3_IGHJ4 (18.2%). We evaluated the binding affinity of the isolated VHH clones to each target antigen using surface plasmon resonance (SPR) with the iMSPR mini-instrument. The results demonstrated that most of the selected VHHs exhibited equilibrium dissociation constant (K_D_) values in the low nanomolar range, indicating strong and specific binding capabilities toward their respective target antigens. Moreover, we assessed the thermal stability of the isolated VHH clones through a protein thermal shift (PTS) assay. The selected VHHs exhibited high thermal stability, with melting temperature (T_m_) values ranging from 60.5 °C to 77.5 °C (Table 1).

## 3. Discussion

The strategic utilization of phage display libraries stands as a crucial approach for identifying and advancing antibodies with precise target specificity. They are categorized into naïve, immune, or synthetic libraries [31,32,33,34,35]. Traditionally, the generation of VHHs involves immunization to establish immune libraries. However, this method is laden with challenges, including the resource-intensive and time-consuming nature of the process [43]. Furthermore, the construction of immune libraries can be hampered by immune tolerance to self-antigens and the relatively low antigenicity of certain targets. This can ultimately lead to reduced immune responses and suboptimal efficiency in selecting antibodies with specific target-binding [44]. The advent of naïve libraries, derived from a large number of primary B cells from non-immunized animals, offers a solution to these limitations [45]. Such libraries circumvent the constraints posed by immune tolerance and antigenicity. Importantly, within naïve libraries, the likelihood of isolating high-affinity binders increases with the library’s size [46,47]. In this study, we established an extensive alpaca-derived single-domain antibody (VHH) library, followed by comprehensive characterization using NGS. To validate the quality of the library, we successfully isolated VHHs with high binding affinity and stability for three therapeutically relevant antigens: programmed cell death ligand 1 (PD-L1) and the receptor-binding domains (RBDs) of the SARS-CoV-2 delta (B.1.617.2) and omicron (BA.2) variants. These findings suggest that the diverse and natural VHH antibody repertoire of the library holds promise for advancing VHH-based therapeutic research, including the development of humanized synthetic VHH libraries.

Sequencing-based analysis is essential for assessment of the library to evaluate the diversity and character of the library after construction. Traditionally, antibody libraries have been analyzed by isolating 10^2^–10^3^ clones using Sanger sequencing [48]. However, this low-throughput analysis method only represents a very small portion of the actual library. Therefore, NGS, a high-throughput method, is considered to be a useful technology for overcoming these limitations [42]. NGS technology allows an analysis of the library’s quality with high throughput and a relatively low cost [41]. Several previous studies have evaluated the quality of naïve, immune, or synthetic VHH libraries using NGS after construction. However, these libraries typically have been analyzed using an average of approximately 10^5^ sequences via NGS [49,50,51]. While this quantity may be sufficient to predict the library’s diversity, increasing the number of analyzed sequences further enhances the statistical accuracy. In this study, a naïve library derived from 20 non-immunized alpacas was evaluated through an extensive sequence analysis with 5,648,592 read counts. Our NGS results, based on vast amounts of sequence data, can provide greater insights into the VHH’s immune repertoire in nature, providing valuable information for further VHH research.

Antibody libraries serve as an invaluable resource for the isolation of antibodies with diverse specificities and functionalities, playing a crucial role in advancing therapeutic research, diagnostic applications, and the development of targeted treatments [52,53]. Our study highlights the extensive VHH repertoire within the constructed library, showing a variety of VHH sequences. Several lines of evidence support this notion. First, we utilized VHH genes from 1.6 × 10^9^ lymphocytes of 20 alpacas, enhancing the genetic diversity compared with the typical naïve libraries of VHHs constructed from 10^7^ to 10^8^ lymphocytes [50,54,55,56]. Second, the constructed library comprised 7.16 × 10^10^ antibody clones, with approximately 92% being unique sequences. This vast size and extensive collection enhanced the library’s potential diversity compared with previous phage display libraries, which typically range in size from 10^7^ to 10^9^ [56,57,58,59,60]. In addition, high-affinity antibodies are more successfully isolated from larger libraries compared with smaller ones [61,62,63]. Consequently, the substantial size of our library (7.16 × 10^10^) increases the likelihood of isolating high-affinity target-specific antibodies through phage display technology. Third, the CDR3 exhibited a diverse range of lengths, from 5 to 31 in the constructed library and from 8 to 19 in selected VHHs, which is crucial for forming various tertiary structures within the CDRs, contributing to the diversity of paratopes and the overall versatility of the library [64]. Fourth, the frequency of non-germline amino acids in the CDR1 and CDR2 of the constructed library provided insights into the frequency of somatic mutations in the library. Our NGS results revealed that only 0.92% of the VHH sequences lacked non-germline amino acids in CDR1 and CDR2, while 82% contained five or more non-germline amino acids. Fifth, the mutation analysis revealed a higher frequency of somatic mutations in the constructed library compared with the previous analysis of the VHH’s repertoire. Unlike the prior analysis, where mutation ratios were below 33% in CDR1 and CDR2, the constructed library exhibited mutation frequencies of 45.1% and 44.3% in CDR1 and CDR2, respectively [50]. These increased somatic mutation rates significantly contribute to the genetic diversity of antibodies. Sixth, CDR1 and CDR2 in the constructed library exhibited significantly higher diversity compared with the reference germline sequences in the IMGT alpaca antibody database, ranging from 4.3- to 24.8-fold. Lastly, to demonstrate the library’s feasibility, we successfully isolated VHHs against three therapeutically relevant antigens: PD-L1 and the RBDs of the SARS-CoV-2 delta (B.1.617.2) and omicron (BA.2) variants through phage display-based biopanning. This validated the potential of the high-quality alpaca VHH library for selecting antibodies with high-affinity binding to the target antigens and its capability to isolate multiple antibodies by accessing new epitopes of three therapeutically relevant antigens, showcasing its versatile utility.

The biophysiochemical properties of antibodies, particularly their stability and affinity, are pivotal for the effective development of therapeutic antibodies. Despite the demonstrated efficacy of mAbs with high affinity and stability, their large molecular size limits their penetration of tissue and production [2]. To address these challenges, researchers have attempted to develop therapeutic antibodies with smaller single-chain variable fragment (scFv) forms [65]. However, these efforts have faced limitations, such as reduced thermal stability [66,67]. Alternatively, VHHs are known to have improved thermal stability despite their smaller size compared with scFvs, making them a promising candidate for therapeutic antibodies [68,69]. Tadokoro et al. measured the thermal stability of 10 approved antibodies in scFv form, with a T_m_ ranging from 52.3 °C to 71.2 °C [67]. In comparison, selected VHHs obtained through biopanning from the constructed library exhibited a T_m_ range of 60.5 °C to 77.5 °C, with an average of 68.7 °C. These results indicate that the selected VHHs have higher thermal stability compared with scFvs. Furthermore, the selected VHHs exhibited favorable affinity, with K_D_ values in the low nanomolar range. Previously reported monovalent VHHs showed a wide range of affinities from the picomolar to micromolar levels. Notably, caplacizumab, a bivalent form of an United States Food and Drug Administration (US FDA)-approved therapeutic VHH antibody, exhibits an affinity of 1 nM in its monovalent form [70]. Additionally, US FDA-approved antibodies such as nivolumab, daratumumab, and rituximab have affinities in the range of 4.03 nM, 4.36 nM, and 8.0 nM, respectively [71,72,73]. Given that bivalency enhances an antibody’s avidity, the nanomolar-level affinities of the monovalent VHHs discovered in this study underscore the potential of our library to identify therapeutically relevant antibodies. Collectively, our selected VHHs verified that the constructed library is a valuable tool for discovering VHHs with promising characteristics for therapeutic applications, including small size, high affinity, and high stability.

In conclusion, our endeavor led to the construction of an extensive alpaca VHH library that is rich in diverse antibody repertoires and is compatible with various antibody selection techniques, including phage display technology. The diversity of this library was substantiated through a comprehensive analysis leveraging NGS. Importantly, our work demonstrated the library’s capability to efficiently isolate antibodies tailored to specific target antigens. These antibodies selected from our library exhibited high thermal stability and strong affinity for target antigens closely associated with cancers and infectious diseases. The wide range of natural VHH antibodies in the library provides valuable insights for VHH therapeutic research, paving the way for the generation of humanized synthetic VHH libraries and breakthroughs in VHH-based treatments. Furthermore, identification of the antibodies’ epitope can provide valuable insights into their binding specificity and mechanism of action. As the next step, we plan to perform epitope mapping of selected antibodies using high-density peptide arrays. In the near future, we hope to utilize this versatile resource to develop novel diagnostic and therapeutic options for a variety of diseases.

## 4. Materials and Methods

### 4.1. Construction of an Alpaca Single-Domain Antibody (VHH) Library

In total, 1.6 × 10^9^ lymphocytes were obtained from 1 L of non-immunized alpaca blood and were purchased from AlpaLife (Shenzhen, China). The lymphocytes were lysed with TRIzol reagent, followed by the addition of 0.2 mL of chloroform and incubation at −20 °C for 20 min to extract total RNA. After centrifugation at 12,000× *g* for 20 min, the aqueous phase containing the total RNA was obtained. The total RNA was then precipitated through the addition of 0.6 mL of isopropyl alcohol. After incubation on ice for 10 min and centrifugation at 12,000× *g* for 10 min, the resulting pellet was washed with 70% ethanol. Finally, the concentration of total RNA dissolved in DNase-free and RNase-free water (Invitrogen, Waltham, MA, USA) was measured using a NanoDrop^TM^ 2000/2000c Spectrophotometer (Thermo Fisher Scientific, Waltham, MA, USA).

First-strand cDNA was synthesized from 5 µg of the total RNA template through reverse transcription using oligo-dT and the SuperScript^TM^ III First-Strand Synthesis System (Invitrogen). PCR was then performed to amplify each VHH gene of the IgG2 and IgG3 types, using Q5 High-Fidelity DNA Polymerase (New England Biolabs, Ipswich, MA, USA), Pfu DNA polymerase (Promega, Madison, WI, USA), or the Platinum SuperFi II PCR Master Mix (Invitrogen), along with 2 µL of the cDNA template and primer sets designed to target only VHH genes containing Sfi I sites (5′-GGC-CCA-GGC-GGC-C-3′ and 5′-GGC-CAG-GCC-GGC-C-3′). The VHH’s PCR products were visualized on a 0.8% (*w*/*v*) agarose gel and purified using the QIAquick Gel Extraction Kit (Qiagen, Hilden, Germany).

Next, the VHH’s PCR products were digested with Sfi I (New England Biolabs) and cloned into the pComb3XSS phagemid vector. The ligated products were transformed into competent *E. coli* ER2738 cells through electroporation using a MicroPulser Electroporator (Bio-Rad Laboratories, Hercules, CA, USA). An aliquot of 100 µL was serially diluted and plated on LB-carbenicillin agar plates to measure the transformation titer. The transformed ER2738 cells were cultured in 4 L of super broth (SB) media (3% (*w*/*v*) tryptone, 2% (*w*/*v*) yeast extract, and 1% (*w*/*v*) MOPS; pH 7.0), supplemented with 50 µg/mL carbenicillin and 1% (*w*/*v*) glucose, at 37 °C in a shaking incubator until the optical density at 600 nm (OD600) reached 1.0. Following centrifugation at 5000 rpm for 7 min, the cell pellet was resuspended in 60 mL of the SB media with 10% glycerol and divided into 1 mL aliquots, which were then stored at −80 °C.

### 4.2. Next-Generation Sequencing of the Antibody Library

The VHH antibody library was amplified using KAPA HiFi HotStart DNA polymerase (Roche, Basel, Switzerland) following the manufacturer’s protocol. The primer sequences used for amplification are listed in Supplementary Table S6. In total, 120.4 ng of plasmids was used as the template for the PCR reaction. The resulting PCR product was purified using CeleMag^TM^ Clean-up Beads (Celemics, Inc., Seoul, Republic of Korea), and the concentration of the purified product was measured using the TapeStation 4200 instrument with D1000 ScreenTape (Agilent Technologies, Santa Clara, CA, USA).

For the preparation of the NGS library, 50 ng of the purified PCR product was used. The xGen^TM^ NGS Library Preparation kit (Integrated DNA Technologies, Inc., Coralville, IA, USA) was used for this purpose. Briefly, the A-tailing and adapter ligation steps were performed using the Enzymatic Prep Master Mix and Ligation Master Mix, respectively. To enrich the adapter-ligated product, PCR amplification was conducted using the IDT PCR Master Mix with the Index 1 and 2 adapters (Integrated DNA Technologies, Inc., Coralville, IA, USA). The sequences of the index adapters used are listed in Supplementary Table S7.

Subsequently, a quality control procedure was performed on the TapeStation 4200 instrument with D1000 ScreenTape to confirm that the length of the NGS library exceeded 400 bp and that the concentration was over 5 ng/μL. The final NGS library was sequenced on the MiSeq platform using the v3 300PE kit (Illumina Inc., San Diego, CA, USA), providing valuable sequence data for the VHH genes, and enabling further analysis and characterization of the antibody library.

### 4.3. Analysis of the Antibody Library

The raw NGS data obtained from MiSeq sequencing were processed for analysis. The R1 and R2 reads were merged using Paired-End read merger (PEAR) software 0.9.8 (The Exelixis Lab, Heidelberg, Germany) with the default parameters [74]. The merged reads were then filtered on the basis of the presence of the primer sequences used. Only reads containing both primer sequences were retained for further analysis. The filtered reads were aligned to alpaca germline sequences, which were retrieved from the IMGT database, using IgBLAST v1.21.0 and the IMGT’s numbering. Since IgBLAST did not have built-in support for auxiliary data from alpacas, we generated the auxiliary data from the J gene’s information obtained from the IMGT database. Among the aligned sequences, only functional sequences that contained all CDRs and were annotated as ‘productive’ were used for further analysis. The analysis included examining germline usage, determining the length of CDR3, and analyzing the amino acid composition of the CDRs and non-germline amino acids.

### 4.4. Rescue of the VHH-Displaying Phage

To rescue the VHH-displaying phage, the working stock of the single-domain antibody (VHH) library was cultured in 1 L of SB media, which contained 3% (*w*/*v*) tryptone, 2% (*w*/*v*) yeast extract, and 1% (*w*/*v*) MOPS at pH 7.0. The media were supplemented with 50 µg/mL carbenicillin, and the culture was incubated in a shaking incubator at 37 °C until the OD600 reached 0.5. Once the OD600 reached the desired value, the cultured VHH library was mixed with the VCSM13 helper phage at a ratio of 1:5 (library: helper phage). The mixture was then incubated in a shaking incubator at 37 °C for 2 h. Following this incubation, kanamycin was added to the mixture at a final concentration of 70 μg/mL, and the culture was further incubated in a shaking incubator at 37 °C for 18 h. After the incubation period, the phage particles were separated from the culture’s supernatant by centrifugation at 8000× *g* for 40 min. To the resulting supernatant, 4% (*w*/*v*) PEG8000 and 3% (*w*/*v*) NaCl were added, and the mixture was incubated on ice for 1 h. Subsequently, the mixture was centrifuged at 11,000× *g* for 50 min, and the supernatant was discarded. The resulting phage pellet was retained.

To further purify the phage, the pellet was resuspended in 3 mL of 3% (*w*/*v*) bovine serum albumin (BSA) in phosphate-buffered saline (PBS) and centrifuged at 11,000 rpm for 5 min to remove cellular debris. The supernatant was then filtered through a 0.2 µm filter to obtain the final VHH-displaying phage. This purified phage preparation was used for downstream applications, such as screening for specific binding antibodies or performing further functional assays.

### 4.5. Selection of VHHs from a Phage Display Antibody Library

Biopanning was performed to isolate VHH antibodies specific to PD-L1 and the RBDs of the SARS-CoV-2 delta (B.1.617.2) and omicron (BA.2) variants from the constructed library. For the selection of antigen-specific VHH, 4 µg of recombinant human PD-L1 (rhPD-L1) protein (Sino Biological, Beijing, China), SARS-CoV-2 B.1.617.2 RBD protein (Sino Biological), and SARS-CoV-2 BA.2 RBD protein (ACROBiosystem, Newark, DE, USA) were individually coated onto magnetic beads (Dynabeads M-270 Epoxy; Invitrogen) according to the manufacturer’s instructions. The coated beads were then washed three times with PBS containing 0.05% (*v/v*) Tween 20 (0.05% PBST) and blocked with 3% BSA in 0.05% PBST, rotating for 2 h at room temperature (RT). Subsequently, five rounds of biopanning were performed to select VHHs specific to PD-L1 and the RBD of SARS-CoV-2 omicron (BA.2), while four rounds were conducted for the RBD of SARS-CoV-2 delta (B.1.617.2). In each round, 10^12^ colony-forming units of VHH-displaying phages were incubated with blocked beads for 2 h with rotation at RT. Next, unbound phages were washed with PBS containing 0.1% (*v/v*) Tween 20 (0.1% PBST), and the number of washing steps was gradually increased in each round. For PD-L1, the magnetic beads were washed once in the first round, three times in the second round and five times in the third, fourth, and fifth rounds. For the RBD of SARS-CoV-2 delta (B.1.617.2), the magnetic beads were washed 3 times in the first round, 5 times in the second round, and 10 times in the third and fourth rounds. For the RBD of SARS-CoV-2 omicron (BA.2), the magnetic beads were washed 3 times in the first round, 5 times in the second round, and 10 times in the third, fourth, and fifth rounds. After the washing step, the antigen-binding phages were eluted with 0.1 M glycine (pH 2.6), followed by neutralization with 1 M Tris-HCl (pH 8.9). Phage recovery and amplification were performed with helper phages (VCSM13) after each round of biopanning to increase the number of target-specific phages, as previously described [75].

### 4.6. Phage ELISA

For phage ELISA, 96 colonies were randomly selected and inoculated into 1 mL of SB media supplemented with 50 μg/mL of carbenicillin in 96-deep-well plates (Axygen, Union City, CA, USA) and incubated at 37 °C for 6 h to allow the production of phages. After the incubation, 10^9^ plaque-forming units of VCSM13 and 70 μg/mL of kanamycin were added to the plates, and the mixture was incubated overnight at 37 °C. The plates were then centrifuged at 3000× *g* for 30 min to separate the phages’ supernatant. Ninety-six-well high-binding microplates (Corning, NY, USA) were coated with 0.1 μg of recombinant human PD-L1 (rhPD-L1) protein (Sino Biological), SARS-CoV-2 B.1.617.2 RBD protein (Sino Biological), and SARS-CoV-2 BA.2 RBD protein (ACROBiosystem), and incubated overnight at 4 °C. After blocking with 3% (*w*/*v*) BSA in PBS, the plates were incubated with 100 μL of the phage’s supernatant at 37 °C for 2 h, allowing the VHH-displaying phages to bind to their respective target antigens. Following incubation, the plates were washed thrice with 0.05% PBST to remove unbound phages. HRP-conjugated anti-M13 antibody (1:5000; Sino Biological) was then added, and the plates were incubated at 37 °C for 1 h to detect the bound phages. After three washes with 0.05% PBST, a 3,3′,5,5′-tetramethylbenzidine (TMB) substrate solution (Thermo Fisher Scientific, Waltham, MA, USA) was added for colorimetric detection. The reaction was allowed to proceed, and the absorbance was measured at 450 nm using a microtiter plate reader (Bio-Tek Instruments, Winooski, VT, USA).

### 4.7. Production and Purification of Selected VHHs

*E. coli* BL21 (DE3) colonies containing the pComb3XSS phagemid vector encoding VHH genes were cultured in 3 mL of SB media with 50 µg/mL carbenicillin at 37 °C overnight. This initial culture was then transferred to 400 mL of fresh SB media supplemented with 50 µg/mL carbenicillin and incubated at 37 °C until reaching an OD600 value of 0.5. Isopropyl-β-D-thiogalactopyranoside (Bio Basic Inc., Markham, ON, Canada) was added to a final concentration of 1 mM, and the induced cells were grown at 37 °C for 4 h. Following centrifugation at 4000× *g* for 15 min, a periplasmic extract was obtained using the osmotic shock method. The cell pellets were resuspended with 15 mL of an ice-cold 1× TES buffer (50 mM Tris-HCl, 1 mM EDTA, 20% (*w*/*v*) sucrose, pH 8.0), and 22.5 mL of 0.2× TES buffer was added subsequently. After incubation on ice for 30 min, the suspension was centrifuged at 6000× *g* for 20 min, yielding the resulting supernatant. The periplasmic extract was incubated with 0.2 mL of HisPur™ Ni-NTA resin (Thermo Fisher Scientific) for 1 h at 4 °C with gentle rotation. The mixture was loaded onto a 2 mL polypropylene column (Thermo Fisher Scientific), and the agarose beads were washed with 2 mL of the washing buffer (20 mM Tris-HCl, 200 mM NaCl, and 5 mM imidazole; pH 7.5). The VHH was eluted using two 0.5 mL fractions of the elution buffer (20 mM Tris-HCl, 200 mM NaCl, and 500 mM imidazole; pH 7.5).

To remove imidazole from the protein solution and exchange the buffer with a physiological buffer, dialysis was conducted in PBS using Slide-A-Lyzer dialysis cassettes (3500 Da MWCO, Thermo Fisher Scientific). The resulting VHHs were then ready for further analysis using SPR.

### 4.8. Determination of the Binding Kinetics of Selected VHHs

The binding kinetics of selected VHHs were analyzed at RT on an iMSPR mini-instrument (iCLUEBIO, Seongnam, Republic of Korea) using an HBST buffer (10 mM HEPES, 150 mM NaCl, and 0.005% (*v/v*) Tween-20; pH 7.4) as a running buffer. The recombinant human PD-L1 (rhPD-L1) protein (Sino Biological), SARS-CoV-2 BA.2 RBD protein (ACROBiosystem), and SARS-CoV-2 B.1.617.2 RBD protein (Sino Biological) were covalently immobilized on the surface of a COOH–Au chip (iCLUEBIO) through standard amine coupling. Serially diluted concentrations of the selected VHHs were injected onto the surface of a sensor chip at a flow rate of 30 μL/min. The association phase lasted for 2 min, followed by a 5 min dissociation phase. Regeneration of the sensor chips after each cycle was achieved by injecting glycine-HCl (pH 2.4) to remove bound antibodies from the sensor chip’s surface. The K_D_ values were calculated using the iMSPR’s analytical software (TraceDrawer 1.9.2; iCLUEBIO).

## Figures and Tables

**Table 1:** Biochemical characterization of the selected single-domain antibodies specific to three therapeutically significant antigens.

Antigen	Antibody (VHH)	K_a_ (1/Ms)	K_d_ (1/s)	K_D_ (nM)	T_m_ (°C)	CDR3 Length	Germline V Gene	Germline J Gene
Human PD-L1	K113.1	2.36 × 10^4^	9.36 × 10^−5^	3.96	66.7	8	IGHV3S53	IGHJ4
	K113.2	1.48 × 10^5^	9.23 × 10^−4^	6.22	76.4	8	IGHV3S53	IGHJ4
SARS-CoV-2 B.1.617.2 RBD	K114.1	1.96 × 10^5^	1.41 × 10^−3^	7.19	68.3	19	IGHV3-3	IGHJ4
	K114.2	2.30 × 10^5^	2.53 × 10^−3^	11.0	66.4	19	IGHV3-3	IGHJ4
	K114.3	3.57 × 10^5^	3.56 × 10^−3^	9.98	62.4	9	IGHV3S1	IGHJ6
	K114.4	1.19 × 10^5^	4.24 × 10^−3^	35.7	64.1	9	IGHV3S1	IGHJ6
	K114.5	2.87 × 10^5^	6.54 × 10^−3^	22.8	68.8	13	IGHV3S53	IGHJ6
	K114.6	3.60 × 10^5^	2.98 × 10^−3^	8.28	60.5	9	IGHV3S1	IGHJ6
SARS-CoV-2 BA.2 RBD	K115.1	2.80 × 10^5^	2.66 × 10^−3^	9.21	71.6	12	IGHV3S53	IGHJ4
	K115.2	2.57 × 10^5^	4.20 × 10^−4^	1.63	72.9	17	IGHV3-3	IGHJ6
	K115.3	1.53 × 10^5^	6.86 × 10^−4^	4.48	77.5	12	IGHV3S53	IGHJ4

K_a_, association constant; K_d_, dissociation constant; K_D_, equilibrium dissociation constant; T_m_, melting temperature.

## Data Availability

The datasets used and/or analyzed during the current study are available from the corresponding author on reasonable request.

## Supplementary Materials

The supporting information can be downloaded at https://www.mdpi.com/article/10.3390/ijms25094791/s1.
