# Supplementary material for: Development, High-Throughput Profiling, and Biopanning of a Large Phage Display Single-Domain Antibody Library
Source: Int J Mol Sci. 2024 Apr 27;25(9):4791. doi: 10.3390/ijms25094791 (PMC11083953; doi:10.3390/ijms25094791)
Supplement: Supplementary file 1 [file ijms-25-04791-s001.zip › ijms-2948687-supplementary.pdf]

Development, High-Throughput Profiling, and Biopanning of a Large Phage Display Single-Domain Antibody Library

Hee Eon Lee [1,†], Ah Hyun Cho [1,†], Jae Hyeon Hwang [1], Ji Woong Kim [1], Ha Rim Yang [1], Taehoon Ryu [2], Yushin Jung [2] and Sukmook Lee [1,3,4*]

[1] Department of Biopharmaceutical Chemistry, Kookmin University, Seoul 02707, Republic of Korea
[2] ATG Lifetech Inc., Seoul 08507, Republic of Korea
[3] Department of Applied Chemistry, Kookmin University, Seoul 02707, Republic of Korea
[4] Antibody Research Institute, Kookmin University, Seoul 02707, Republic of Korea
* Correspondence: lees2018@kookmin.ac.kr; Tel.: +82-2-910-6763
† These authors contributed equally to this work

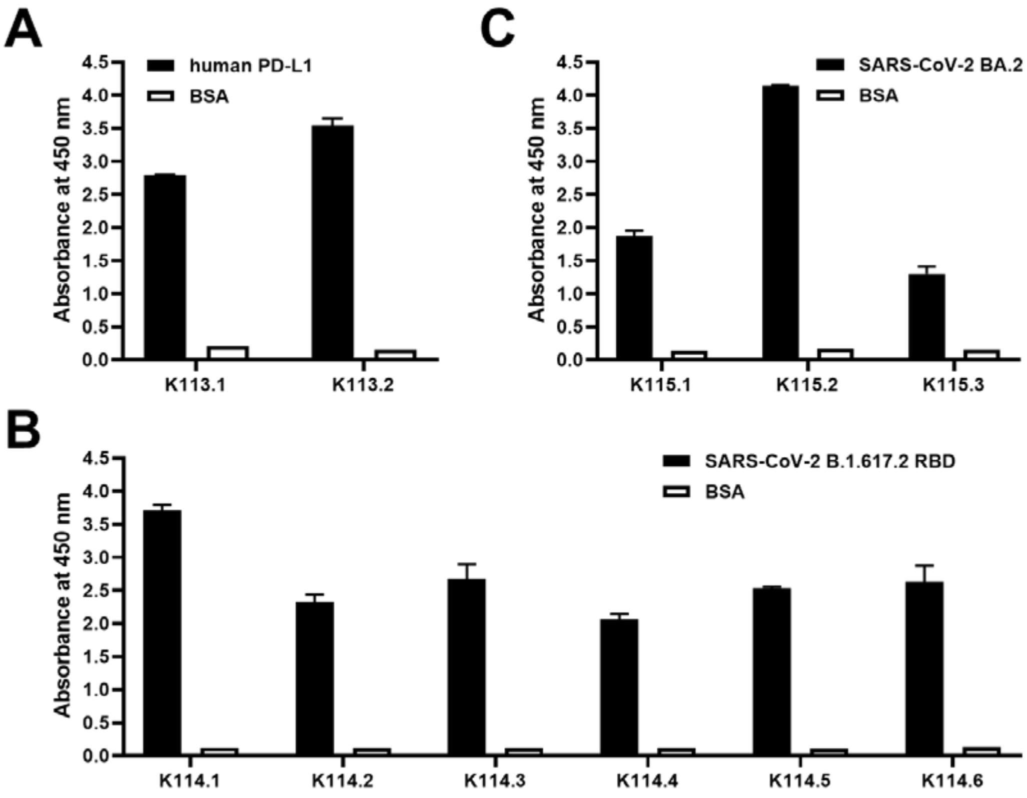

Supplementary Figure S1. Specific binding of the selected VHHs to target antigens. Phage ELISA was performed to verify the reactivity of the selected VHHs to human PD-L1 (A), SARS-CoV-2 BA.2 RBD (B), and SARS-CoV-2 B.1.617.2 RBD (C). Bovine serum albumin (BSA) was used as a negative control.

Supplementary Table S1. Primers used for the construction of the single domain antibody library.

Primer name	5′ to 3′ Sequence
**Alp-Vh-F1 Sfi I**	ACTGTGGCCCAGGCGGCCCAGKTGCAGCTCGTGGAGTCNGGNGG
**AlpVhh-R1 Sfi I**	ATGACTCGCGGCCGGCCTGGCCTCGTGGGGGTCTTCGCTGTGGTGCG
**AlpVhh-R2 Sfi I**	ATGACTCGCGGCCGGCCTGGCCTCGCCTTGTGGTTTTGGTGTCTTGGG

Supplementary Table S2. The percentage of pairings between the 63 individual IGHV gene segments and the seven IGHJ gene families in the constructed library.

	IGHJ1	IGHJ2	IGHJ3	IGHJ4	IGHJ5	IGHJ6	IGHJ7
**IGHV1-1**	0.000	0.000	0.000	0.000	0.000	0.000	0.000
**IGHV3-1**	0.000	0.000	0.000	0.000	0.000	0.000	0.000
**IGHV3-2**	0.000	0.008	0.002	0.139	0.000	0.028	0.026
**IGHV3-3**	0.000	0.028	0.015	5.149	0.002	0.455	0.195
**IGHV3S1**	0.000	0.017	0.029	0.475	0.000	0.112	0.135
**IGHV3S10**	0.000	0.000	0.000	0.000	0.000	0.000	0.000
**IGHV3S12**	0.000	0.001	0.000	0.009	0.000	0.003	0.008
**IGHV3S14**	0.000	0.000	0.000	0.000	0.000	0.000	0.000
**IGHV3S17**	0.000	0.001	0.000	0.010	0.000	0.002	0.001
**IGHV3S18**	0.000	0.000	0.000	0.000	0.000	0.000	0.000
**IGHV3S2**	0.000	0.000	0.000	0.000	0.000	0.000	0.000
**IGHV3S20**	0.000	0.000	0.000	0.000	0.000	0.000	0.000
**IGHV3S22**	0.000	0.000	0.000	0.000	0.000	0.000	0.000
**IGHV3S24**	0.000	0.000	0.000	0.000	0.000	0.000	0.000
**IGHV3S25**	0.000	0.005	0.008	0.508	0.015	0.119	0.062
**IGHV3S26**	0.000	0.000	0.000	0.000	0.000	0.000	0.000
**IGHV3S27**	0.000	0.000	0.000	0.000	0.000	0.000	0.000
**IGHV3S28**	0.000	0.002	0.006	0.429	0.001	0.061	0.050
**IGHV3S29**	0.000	0.000	0.004	0.010	0.000	0.001	0.001
**IGHV3S30**	0.000	0.007	0.028	1.420	0.003	0.628	0.141
**IGHV3S31**	0.000	0.000	0.000	0.001	0.000	0.000	0.000
**IGHV3S32**	0.000	0.000	0.000	0.001	0.000	0.000	0.000
**IGHV3S33**	0.000	0.000	0.000	0.010	0.000	0.007	0.004
**IGHV3S34**	0.000	0.001	0.000	0.025	0.000	0.031	0.001
**IGHV3S35**	0.000	0.000	0.000	0.000	0.000	0.000	0.000
**IGHV3S36**	0.000	0.000	0.000	0.000	0.000	0.000	0.000
**IGHV3S37**	0.000	0.001	0.031	0.095	0.000	0.014	0.005
**IGHV3S38**	0.000	0.000	0.000	0.000	0.000	0.000	0.000
**IGHV3S39**	0.000	0.045	0.043	0.931	0.001	0.213	0.213
**IGHV3S40**	0.000	0.000	0.000	0.007	0.000	0.002	0.000
**IGHV3S41**	0.000	0.031	0.112	2.533	0.002	0.368	0.203
**IGHV3S42**	0.000	0.000	0.000	0.000	0.000	0.000	0.000
**IGHV3S43**	0.000	0.000	0.000	0.000	0.000	0.000	0.000
**IGHV3S44**	0.000	0.000	0.000	0.000	0.000	0.000	0.000
**IGHV3S45**	0.000	0.000	0.000	0.000	0.000	0.000	0.000
**IGHV3S5**	0.000	0.000	0.000	0.000	0.000	0.000	0.000
**IGHV3S53**	0.003	1.369	0.566	41.481	0.670	5.979	4.217
**IGHV3S54**	0.000	0.017	0.007	0.052	0.005	0.016	0.003
**IGHV3S55**	0.000	0.000	0.000	0.044	0.000	0.013	0.002
**IGHV3S56**	0.000	0.000	0.000	0.074	0.000	0.012	0.000
**IGHV3S57**	0.000	0.000	0.000	0.008	0.000	0.001	0.001
**IGHV3S58**	0.000	0.000	0.000	0.007	0.000	0.000	0.000
**IGHV3S59**	0.000	0.000	0.000	0.000	0.000	0.000	0.000
**IGHV3S6**	0.000	0.000	0.000	0.046	0.000	0.004	0.014
**IGHV3S60**	0.000	0.001	0.000	0.494	0.000	0.016	0.010
**IGHV3S61**	0.000	0.177	0.108	5.684	0.004	2.363	1.259
**IGHV3S62**	0.000	0.002	0.001	0.088	0.000	0.035	0.017
**IGHV3S63**	0.000	0.001	0.001	0.107	0.000	0.016	0.022

**IGHV3S64**	0.000	0.000	0.000	0.000	0.000	0.000	0.000
**IGHV3S65**	0.001	0.315	0.114	11.964	0.022	3.663	2.535
**IGHV3S66**	0.000	0.000	0.000	0.019	0.000	0.004	0.017
**IGHV3S67**	0.000	0.000	0.000	0.000	0.000	0.000	0.000
**IGHV3S68**	0.000	0.000	0.000	0.019	0.000	0.000	0.000
**IGHV3S7**	0.000	0.001	0.001	0.036	0.000	0.006	0.004
**IGHV3S8**	0.000	0.000	0.000	0.022	0.000	0.009	0.018
**IGHV3S9**	0.000	0.001	0.000	0.083	0.000	0.091	0.012
**IGHV4S1**	0.000	0.024	0.017	0.238	0.000	0.078	0.112
**IGHV4S11**	0.000	0.000	0.000	0.000	0.000	0.000	0.000
**IGHV4S2**	0.000	0.000	0.000	0.000	0.000	0.000	0.000
**IGHV4S5**	0.000	0.014	0.001	0.153	0.000	0.048	0.039
**IGHV4S8**	0.000	0.000	0.000	0.000	0.000	0.000	0.000
**IGHV4S9**	0.000	0.000	0.000	0.000	0.000	0.000	0.000

The percentages of all the pairing of germline V and J gene segments with a cut-off ≥ 0.001 are shown. Pairing with a of the gene segment cut-off < 0.001 are expressed as 0.000.

Supplementary Table S3. The amino acid composition of CDR1, CDR2, and CDR3 in the constructed library, expressed as a percentage.

	**Amino acid composition (%)**																			
	**G**	**A**	**L**	**M**	**F**	**W**	**K**	**Q**	**E**	**S**	**P**	**V**	**I**	**C**	**Y**	**H**	**R**	**N**	**D**	**T**
**CDR1**	13.0	6.7	5.1	0.8	12.5	0.4	0.7	0.5	1.3	13.3	1.2	3.2	7.7	0.1	9.5	1.3	4.3	4.8	5.0	8.7
**CDR2**	18.4	3.2	1.4	0.9	1.0	0.7	1.1	0.3	1.0	18.5	1.6	2.0	13.1	0.2	0.8	0.6	5.5	4.4	6.0	19.5
**CDR3**	9.7	10.6	5.4	1.3	2.8	2.1	1.8	1.9	4.0	7.3	4.5	5.0	2.5	1.9	9.9	2.0	8.3	5.8	8.0	5.4

Supplementary Table S4. The amino acid frequency at each position in CDRs of the constructed library, expressed as a percentage.

Position	G	A	L	M	F	W	K	Q	E	S	P	V	I	C	Y	H	R	N	D	T
**27**	85.38	2.06	0.18	0.03	0.01	0.01	0.61	0.23	3.16	0.55	0.14	0.83	0.15	0.01	0.00	0.00	5.24	0.03	0.48	0.89
**28**	3.33	1.07	4.84	1.06	35.69	0.18	0.49	0.10	0.21	25.78	0.81	2.98	6.52	0.33	0.80	0.48	6.72	3.66	1.71	3.23
**29**	1.18	3.98	2.22	1.29	2.43	0.05	0.28	0.14	0.10	9.88	2.20	3.07	23.53	0.02	2.12	0.21	2.33	4.83	1.94	38.20
**30**	0.78	1.07	25.18	0.70	50.65	0.58	0.17	0.09	0.38	6.61	0.77	2.81	4.37	0.14	1.37	0.41	0.76	0.43	1.70	1.04
**31**	1.81	1.63	4.05	0.09	21.21	0.67	0.37	0.49	0.51	15.80	0.86	1.15	4.39	0.18	0.63	0.25	1.09	27.85	3.47	13.51
**32**	3.57	3.66	2.52	0.39	2.35	0.02	0.08	0.03	2.05	5.10	0.65	37.56	21.86	0.05	1.39	0.16	4.80	0.67	2.55	10.55
**33**	0.34	0.75	1.86	0.38	0.14	0.02	0.16	0.07	0.12	4.13	0.42	1.05	4.81	0.02	2.95	1.20	0.88	58.66	0.84	21.21
**34**	0.83	3.68	7.23	0.20	42.03	4.95	0.14	0.02	0.77	3.24	1.27	1.54	5.80	0.18	3.78	0.40	1.10	0.74	0.66	21.41
**35**	6.90	1.86	0.30	0.53	0.76	0.12	2.02	0.23	4.64	41.82	0.44	1.41	0.82	0.15	0.63	1.04	9.21	5.98	18.47	2.68
**36**	2.09	2.40	5.78	2.39	4.65	0.56	0.66	2.33	0.53	10.29	1.82	3.17	23.47	0.11	12.53	2.21	6.77	7.33	5.49	5.42
**37**	1.45	1.77	1.86	0.19	3.92	0.78	0.91	0.58	0.58	5.32	0.59	1.37	0.82	0.17	57.30	3.74	1.31	12.49	2.90	1.96
**38**	3.73	39.66	0.89	0.19	0.76	0.81	0.36	0.18	0.60	6.66	2.72	10.06	1.71	0.15	1.88	2.05	2.57	2.33	7.70	15.01
**56**	0.04	0.79	3.38	3.03	2.02	0.05	0.33	0.13	0.08	1.69	0.04	3.94	81.84	0.08	0.09	0.12	0.73	0.25	0.06	1.31
**57**	3.74	2.84	1.17	0.85	1.21	0.28	0.77	0.50	0.41	29.22	1.08	1.69	2.93	0.18	0.74	0.60	4.52	8.71	3.69	34.88
**58**	7.21	4.32	1.27	0.63	2.14	4.24	1.94	0.51	1.16	39.77	2.48	1.15	1.67	0.26	1.18	0.89	10.21	8.02	2.37	8.57
**59**	26.32	5.85	1.62	0.41	0.52	0.11	0.82	0.20	1.15	23.45	3.17	1.76	1.26	0.18	0.74	0.50	7.89	5.13	12.18	6.74
**60**	14.30	16.39	1.25	0.43	1.39	0.19	0.38	0.59	0.92	19.78	1.01	1.36	2.08	0.12	0.23	0.79	4.01	2.99	17.79	14.00
**61**	21.13	4.12	0.11	1.41	0.10	0.04	0.78	0.06	20.46	32.47	0.20	1.32	0.41	0.10	0.20	0.35	5.81	3.43	3.42	4.06
**62**	53.02	3.05	0.18	0.19	0.22	0.02	0.13	0.14	2.59	4.52	0.22	1.12	0.26	0.22	0.47	0.28	1.44	2.10	27.52	2.31
**63**	67.40	1.99	0.64	0.22	0.24	0.07	0.28	0.26	1.89	6.11	0.21	1.55	0.75	0.27	0.67	0.81	5.10	1.36	7.94	2.25
**64**	5.52	2.38	1.20	1.22	0.96	0.34	1.95	0.43	0.90	34.16	0.61	3.79	4.91	0.10	2.01	1.13	9.60	8.52	4.21	16.08
**65**	0.19	3.87	0.96	0.20	0.13	0.02	2.19	0.16	0.54	2.72	4.15	0.97	5.38	0.00	0.05	0.02	2.31	0.33	0.12	75.69
**105**	1.50	35.60	0.81	0.15	0.26	0.06	3.26	0.36	0.28	2.55	0.07	1.58	0.21	0.10	3.96	3.87	1.45	41.51	0.39	2.03
**106**	2.74	55.08	2.55	0.44	0.97	0.11	2.82	0.50	0.78	2.55	1.30	7.19	3.01	0.26	0.76	0.21	8.07	0.88	0.44	9.33
**107**	9.02	6.20	3.69	0.71	1.07	1.92	3.35	4.52	8.14	3.03	4.54	6.15	1.93	0.28	2.10	3.75	14.77	3.41	18.62	2.79
**108**	12.47	6.62	8.25	0.73	2.34	2.80	1.69	1.82	4.99	7.97	6.73	6.00	4.62	0.72	5.77	1.73	14.32	2.37	3.15	4.91
**109**	12.51	4.93	6.55	1.21	2.93	2.98	1.44	2.71	2.64	10.49	5.48	6.22	3.50	0.61	5.89	1.31	13.97	2.05	4.96	7.62
**110**	12.72	4.54	8.35	1.00	2.79	2.94	0.95	0.95	1.94	9.47	5.63	9.07	3.70	1.89	6.93	1.62	11.33	2.00	5.31	6.86
**111**	12.49	4.20	7.15	1.06	2.84	3.63	2.13	1.73	1.85	9.66	5.76	6.09	3.17	4.16	8.09	1.59	8.27	3.77	5.18	7.18
**111.1**	11.89	6.76	5.11	0.64	2.04	2.73	1.51	1.81	2.37	11.76	5.70	5.16	3.42	7.56	6.29	1.19	6.08	2.01	4.98	10.98
**111.2**	12.15	4.74	6.60	0.98	1.95	2.24	1.28	1.35	1.95	10.33	5.29	7.10	3.31	7.43	6.71	1.24	8.18	2.05	8.65	6.48
**H111.3**	9.86	4.73	11.05	1.80	2.27	4.17	1.89	2.80	2.29	11.36	3.78	8.41	2.16	5.55	8.84	0.91	4.51	2.70	4.18	6.73
**111.4**	9.85	11.06	3.93	1.72	5.18	1.05	1.37	2.42	2.90	18.06	3.51	5.72	1.65	4.96	6.44	0.89	3.57	3.27	7.94	4.51
**111.5**	4.93	5.70	3.43	3.39	2.91	1.01	1.28	22.63	3.28	6.27	3.13	9.28	2.33	10.41	6.60	0.69	4.62	0.65	4.45	3.03
**111.6**	7.06	4.25	3.06	1.47	0.57	0.37	0.58	1.74	0.90	4.93	2.74	4.33	2.28	2.00	4.21	1.83	2.32	42.53	3.23	9.60
**111.7**	76.84	2.31	1.34	0.56	0.16	0.14	0.17	2.30	0.32	1.10	0.95	1.79	1.35	1.13	0.87	0.28	3.03	0.57	3.62	1.17
**111.8**	71.07	0.24	0.51	0.17	0.07	0.40	0.06	0.36	21.06	0.36	0.14	1.27	0.16	0.12	0.14	0.31	0.31	0.10	1.63	1.52
**111.9**	4.20	4.05	0.15	2.40	3.60	0.30	1.65	47.90	0.30	4.35	4.50	18.02	0.45	0.15	0.60	0.45	4.50	0.15	0.90	1.35
**112.9**	5.60	2.44	5.75	2.09	8.50	0.76	1.53	24.03	1.07	4.84	1.73	19.20	1.02	0.36	0.76	0.05	7.28	2.39	4.63	5.96

**112.8**	2.05	1.45	0.60	0.16	0.33	0.05	0.06	0.66	0.43	0.75	0.58	0.85	0.16	0.44	0.54	0.77	87.07	0.09	0.52	2.44
**112.7**	4.84	3.47	53.66	1.03	0.93	3.29	0.78	0.81	0.47	5.51	1.08	2.41	8.23	3.16	2.96	0.58	1.55	0.79	2.22	2.25
**112.6**	30.54	13.08	3.99	2.13	1.39	0.70	1.15	1.46	1.43	4.60	11.34	4.06	0.88	5.45	4.03	1.58	1.90	0.89	5.40	4.02
**112.5**	4.71	4.13	15.58	4.28	1.28	1.17	1.10	3.15	1.95	7.74	4.36	8.11	3.53	5.20	5.56	0.70	5.06	8.74	3.94	9.71
**112.4**	13.00	4.51	6.10	3.32	1.16	2.82	1.04	1.61	7.09	6.80	3.45	7.17	1.68	10.46	6.30	1.33	4.10	5.08	5.05	7.94
**112.3**	9.07	5.90	8.06	3.83	2.02	5.55	1.19	1.50	2.32	8.12	7.02	5.10	3.70	9.73	8.60	2.01	4.02	1.89	3.68	6.69
**112.2**	11.38	5.81	11.93	1.65	2.35	3.24	3.04	1.08	4.70	8.15	6.26	4.02	2.95	8.42	4.76	1.50	6.75	2.34	4.12	5.56
**112.1**	9.62	5.44	9.14	2.99	2.17	2.54	1.72	2.51	3.78	8.82	7.44	4.72	2.25	2.50	5.34	1.90	8.22	3.23	7.54	8.12
**112**	12.86	8.51	6.07	1.57	2.38	3.14	1.75	2.03	4.28	8.79	12.19	4.41	2.93	1.26	6.05	1.99	7.55	2.28	4.55	5.41
**113**	14.71	5.91	4.37	0.83	1.87	3.95	1.81	1.78	3.94	9.49	4.07	3.82	2.29	0.34	9.99	2.54	11.16	3.59	7.45	6.09
**114**	17.38	5.96	6.58	1.15	3.00	1.45	1.78	4.30	11.61	5.30	6.21	2.96	2.55	0.12	2.94	1.50	8.47	2.50	9.57	4.66
**115**	5.10	2.65	5.40	4.78	12.04	1.73	1.61	1.59	2.83	3.96	3.00	5.00	1.86	0.20	25.85	1.31	11.80	3.20	2.96	3.12
**116**	11.15	3.06	1.13	0.35	0.39	0.51	0.85	1.13	9.55	4.20	1.52	2.27	0.91	0.15	1.20	2.24	2.52	9.99	43.08	3.82
**117**	0.54	3.15	2.75	0.15	5.87	0.61	0.10	0.19	0.17	12.86	0.95	3.50	1.37	0.51	56.67	3.59	0.67	1.99	2.13	2.22

The amino acid frequency at each position in CDRs with a cut-off ≥ 0.01 are represented. Amino acid frequencies with a cut-off < 0.01 are expressed as 0.00.

Supplementary Table S5. Length of complementarity-determining regions (CDR1, CDR2, CDR3) in selected VHHs.

Antigen	Antibody (VHH)	Length		
		CDR1	CDR2	CDR3
**Human PD-L1**	K113.1	8	8	8
	K113.2	8	8	8
**SARS-CoV-2 B.1.617.2 RBD**	K114.1	8	8	19
	K114.2	8	8	19
	K114.3	8	8	9
	K114.4	8	8	9
	K114.5	8	8	13
	K114.6	8	8	9
**SARS-CoV-2 BA.2 RBD**	K115.1	8	8	12
	K115.2	8	8	17
	K115.3	8	7	12

Supplementary Table S6. Primers used for amplification of VHH sequence.

Primer name	5′ to 3′ Sequence
Forward	TGGCTGGTTTCGCTACC
Reverse	TGATGGTGATGGTGCTGG

Supplementary Table S7. Index adapters used for enrichment adapter-ligated product.

Index Adapter	5′ to 3′ Sequence
**Index 1 (i7)**	GATCGGAAGAGCACACGTCTGAACTCCAGTCACNNNNNNNNATCTCGTATGCCGTCTTCTGCTTG
**Index 2 (i5)**	AATGATACGGCGACCACCGAGATCTACACNNNNNNNNACACTCTTTCCCTACACG
